# Reply to Comment on: “Night shift work and risk of breast cancer in women: the Generations Study cohort”

**DOI:** 10.1038/s41416-019-0568-5

**Published:** 2019-09-05

**Authors:** Michael E. Jones, Minouk J. Schoemaker, Emily C. McFadden, Lauren B. Wright, Louise E. Johns, Anthony J Swerdlow

**Affiliations:** 1 Division of Genetics and Epidemiology, The Institute of Cancer Research, London, SW7 3RP UK; 2 Division of Breast Cancer Research, The Institute of Cancer Research, London, SW7 3RP UK; 3 Present Address: Nuffield Department of Primary Care Health Sciences, University of Oxford, Oxford, OX2 6GG UK

**Keywords:** Risk factors, Epidemiology

We thank Kogevinas for his interest in our paper on night-shift work and breast cancer risk in women.^1^ He raises four points:

i. That our question on night work mixes evening and night-shift work, and this would result in the “exposed” group including non-exposed evening workers. This, of course, depends on one’s definition of “exposure”. Interest in night-shift work and breast cancer springs from the hypothesis that exposure to light at night may increase cancer risk.^2^ In 2007, the International Agency for Research on Cancer concluded that there was limited evidence in humans for the carcinogenicity of shift work that involves night work, but overall shift work that involves circadian disruption is probably carcinogenic.^2^ Our analyses included hours 10pm–7am as “exposure” since these involve light at night and may influence the circadian rhythm. The exact times that count as “night” are somewhat arbitrary, if only because nightfall varies by season and latitude, and bedtimes vary between individuals. However, we did present the results for <7 and 7+hours worked per night (Table 2) and found no raised risk with working 7+hours per night. Working 7+hours between 10pm and 7am could not be considered primarily evening work.
ii. That our study includes mostly middle-aged participants and may have misclassified some women who worked night shifts in an earlier period. At recruitment, women in our study had a mean age of 40.2 years, which was considerably younger than the mean ages (between 54 and 59 years) in the five case–control studies in the pooled analysis^3^ cited by Kogevinas as evidence for an association, and much younger than the mean ages in the recent pooling of prospective studies (Million Women Study: 69 years; UK Biobank: 56 years; EPIC-Oxford: 58 years).^4^ To limit the burden on respondents, we did not collect the lifetime history of night shifts but only night work that occurred, or at least ended, during the 10 years before recruitment. Some have suggested that any increased risk associated with night-shift work may diminish soon after exposure ceases,^3,5,6^ and recent, rather than historic, night shifts may be the more appropriate exposure measure. Thus, we analysed night work in the last 10 years, for which period earlier night work would not be the exposure of interest.
iii. That we only included long-duration workers if they had survived and entered the period 10 years before recruitment, and this could bias towards the null analysis by duration. We found no association with duration in our study, and as we acknowledged in our paper, our data for durations beyond 10 years of night work were limited (see response to (ii)).
iv. That the least biased results in our study are certain ones cited by Kogevinas that approached statistical significance. Interpretation of the results, however, needs to consider the whole breadth of the variables analysed, and of the existing literature. We concluded that in the absence of an association between breast cancer and night-shift work overall, or by other measures of dose, duration or intensity in our study, and no evidence for association from the most recent meta-analysis of prospective studies,^4^ our finding of a statistically significant trend with average hours per week on its own did not provide strong support for a real causal association. However, as we noted, unlike traditional carcinogens where cumulative duration and dose response are critical exposures as evidence for the cause–effect association, it is still unclear which exposure “domains” may be important in relation to night-shift work. Therefore, we neither “discarded” the results on intensity, nor selectively focussed on them, but presented them and commented on their material significance in the context of other results and the existing literature.

Finally, with regard to Kogevinas’ advocacy that we should not have published these results, we would note that all epidemiological studies have limitations and are vulnerable to bias. If authors should self-censor information for publication when a study includes potential limitations or biases, as Kogevinas advocates, there would be no literature on shift work. The point of peer review and journal editing is for journals to judge whether papers are of sufficient quality, and for readers to make judgements from the literature overall, including both the significant findings and those that are null, to come to a conclusion about the state of the evidence.

## Data Availability

The data sets generated during and/or analysed during the current study are not publicly available due to confidentiality reasons, but anonymised versions may be available from the corresponding author on reasonable request.
